# Consideration of multiple load cases is critical in modelling orthotropic bone adaptation in the femur

**DOI:** 10.1007/s10237-015-0740-7

**Published:** 2015-11-17

**Authors:** Diogo M. Geraldes, Luca Modenese, Andrew T. M. Phillips

**Affiliations:** 1 Structural Biomechanics, Department of Civil and Environmental Engineering, Skempton Building, Imperial College London, London, UK; 2 Biomechanics Group, Department of Mechanical Engineering, City and Guilds Building, Imperial College London, London, UK; 3 Department of Mechanical Engineering, Sir Frederick Mappin Building, Mappin Street, The University of Sheffield, Sheffield, UK; 4 INSIGNEO Institute for In Silico Medicine, The Pam Liversidge Building, The University of Sheffield, Sheffield, UK

**Keywords:** Femur, Bone adaptation, Orthotropic, Multiple load cases, Finite element modelling, Young’s modulus, Shear modulus, Musculoskeletal, Daily living activities, Bone remodelling, Biomechanics

## Abstract

Functional adaptation of the femur has been investigated in several studies by embedding bone remodelling algorithms in finite element (FE) models, with simplifications often made to the representation of bone’s material symmetry and mechanical environment. An orthotropic strain-driven adaptation algorithm is proposed in order to predict the femur’s volumetric material property distribution and directionality of its internal structures within a continuum. The algorithm was applied to a FE model of the femur, with muscles, ligaments and joints included explicitly. Multiple load cases representing distinct frames of two activities of daily living (walking and stair climbing) were considered. It is hypothesised that low shear moduli occur in areas of bone that are simply loaded and high shear moduli in areas subjected to complex loading conditions. In addition, it is investigated whether material properties of different femoral regions are stimulated by different activities. The loading and boundary conditions were considered to provide a physiological mechanical environment. The resulting volumetric material property distribution and directionalities agreed with *ex vivo* imaging data for the whole femur. Regions where non-orthogonal trabecular crossing has been documented coincided with higher values of predicted shear moduli. The topological influence of the different activities modelled was analysed. The influence of stair climbing on the properties of the femoral neck region is highlighted. It is recommended that multiple load cases should be considered when modelling bone adaptation. The orthotropic model of the complete femur is released with this study.

**Electronic supplementary material:**

The online version of this article (doi:10.1007/s10237-015-0740-7) contains supplementary material, which is available to authorized users.

## Introduction

Osteoporosis weakens bone, increasing the risk of femoral neck fractures which have a concerningly high mortality rate (Goldacre et al. 2002). There is evidence that selected physical activities could increase bone mass density or reduce the rate of bone decay in affected patients (Jamsa et al. 2006; Judex et al. 1997; Judex and Zernicke 2000), particularly in the cortex of the femoral neck region (Allison et al. 2015; Blain et al. 2009), but it is not possible to systematically predict the effect of different activities on local bone quality due to limitations of current experimental and computational techniques.

Adaptation algorithms embedded in finite element (FE) simulations have been used to investigate bone’s response to loading conditions, with bone often assumed to have isotropic material properties, for simplicity (Huiskes et al. 1987). This assumption is insufficient in predicting the directionality of bone’s observed microstructure (Geraldes and Phillips 2014; Skedros and Baucom 2007), a critical factor in understanding bone’s performance and mechanical behaviour (Nazarian et al. 2007). Furthermore, material properties for bone have been measured experimentally and the orthotropic assumption shown to be the closest approximation to bone’s anisotropy, short of a full anisotropic description (Ashman et al. 1984; Cuppone et al. 2004; Turner et al. 1999), presenting a more realistic representation of the femur’s structural behaviour than isotropy (Pidaparti and Turner 1997). Recent work has succeeded in extracting orthotropic material properties from computerised tomography (CT) data, but relies on subjective estimations of regional principal material directions from geometric features or observation of collagen structures amongst volumetric CT data of varying resolution (Blanchard et al. 2013; Yosibash et al. 2008).

Despite achieving physiological material property distribution and directionality, most remodelling algorithms have been applied to study the material property distribution in a portion of the femur, with particular emphasis on the proximal femur, in order to decrease the computational effort required and overcome the difficulty in defining loading conditions for the entire bone (Fernandes et al. 1999; Miller et al. 2002; Tsubota et al. 2009). Few complete continuum femur models have been developed, usually assuming bone to be an isotropic material and not focusing on its adaptation to external loading (Duda et al. 1998; Phillips 2009; Speirs et al. 2007). Therefore, results for all regions and anatomical planes of the femur are not commonly reported.

An orthotropic strain-driven bone adaptation algorithm developed by the authors (Geraldes and Phillips 2014) was applied to a fully balanced FE model of the femur with all muscle and ligament forces included through the use of spring elements, alongside physiological boundary conditions. The model was shown to produce a more physiological material property distribution for the complete femur in comparison with an isotropic modelling approach, whilst simultaneously providing information on the directional properties of the underlying cortical and trabecular structure (Geraldes and Phillips 2014).

Studies have called attention to the function of non-orthogonally intersecting trabeculae in resisting shear stresses and strains resulting from the multitude of load cases bone is subjected to (Garden 1961; Miller et al. 2002; Skedros and Baucom 2007; Tobin 1955). This complex mechanical environment cannot be represented using orthotropic adaptation algorithms under a single load case or a single combined load case, since it would align the material properties with the principal stress directions, resulting in negligible shear resistance as found in Geraldes and Phillips (2014). Multiple instances of activities of daily living need to be considered in order to physiologically reproduce the adaptation of trabecular architecture to complex loading (Carter et al. 1989; Miller et al. 2002; Skedros and Baucom 2007). In an attempt to understand bone’s resistance to shear, a shear modulus component was included in the proposed adaptation algorithm. It is hypothesised that: (1) low shear moduli occur in areas of bone that are simply loaded and high shear moduli occur in areas subject to complex loading conditions and (2) the material properties of different topological regions of the femur are stimulated by different activities. The topological influence of multiple load cases in determining the converged material properties (Young’s and shear moduli) was assessed, and the resulting continuum representation compared with imaging data of femoral cortical and trabecular structure. To the authors’ knowledge, this is the first time orthotropic material properties and directionality have been predicted for a complete 3D model of a bone using a mechanical loading environment incorporating multiple daily living activities. The resulting continuum heterogeneous orthotropic finite element model of the femur is made available and can be downloaded from http://figshare.com/articles/Orthotropic_Femur_Model/1419589.^1^

## Methods

### The finite element femur model

**Fig. 1:**
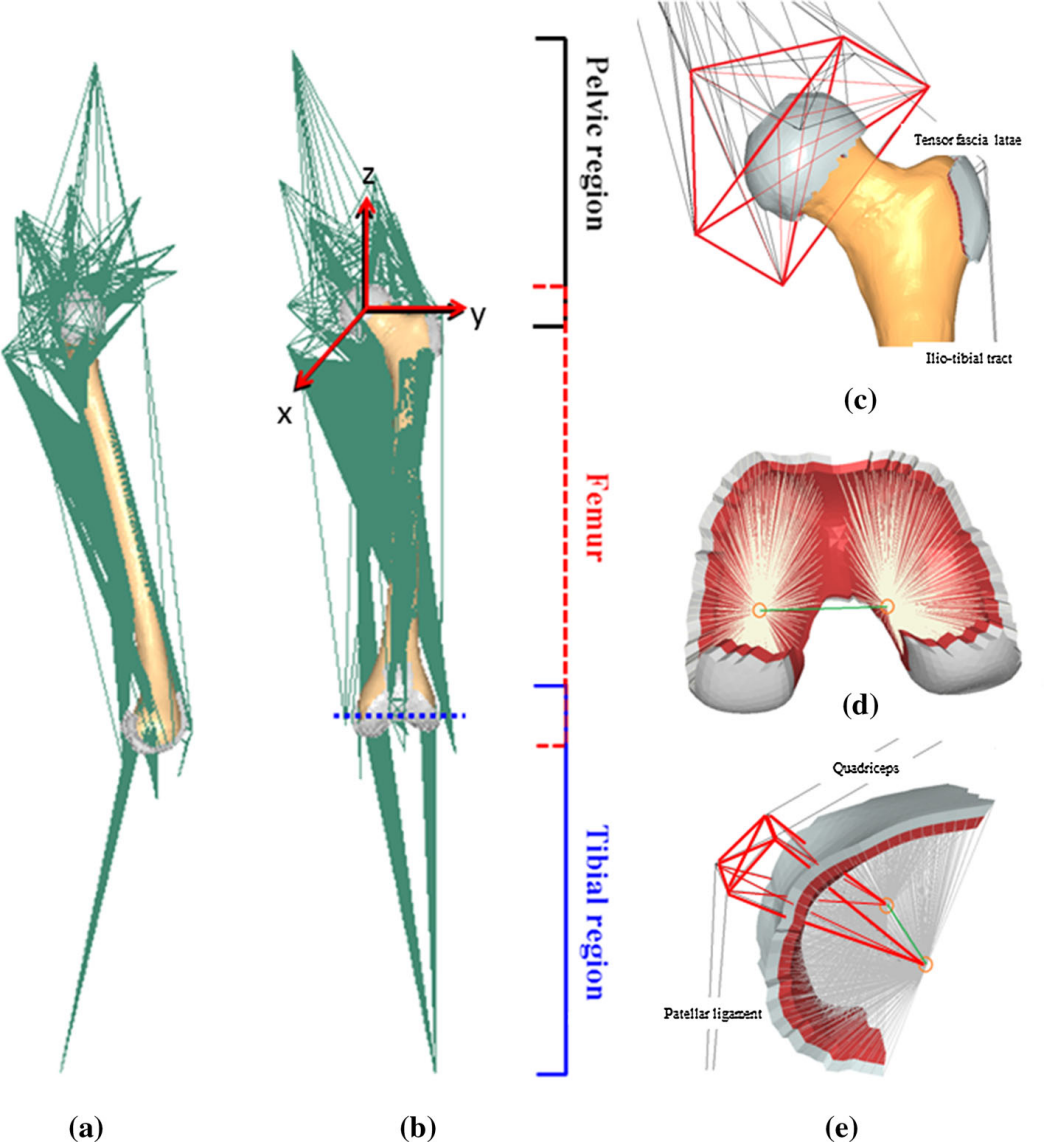
Medial (**a**) and anterior (**b**) views of the finite element model of the whole femur, pelvic (*black*) and femoral (*red*) coordinate systems and functional knee axis (*dashed blue line*). Twenty-six muscles, 7 ligaments (*green*) and load-transfer structures (*grey*) were explicitly included at **c** the hip joint and tensor fascia latae, **d** tibiofemoral joint and **e** patellofemoral joint

The femur model used in this study is modified from that previously described in Geraldes and Phillips (2014), with geometry extracted from the muscle standardised femur (Viceconti et al. 2003). A brief description is given here for completeness. A FE model of the lower limb was created, with the local coordinate systems of the segments defined according to the standards proposed by the International Society of Biomechanics (Wu et al. 2002; Fig. 1).

Twenty-six muscles and seven ligamentous structures (Fig. 1, in green) were represented as groups of spring elements, in number proportional to their insertion area. Musculotendon stiffness was calculated as in Phillips (2009) and Philips et al. (2007) based on the dimensionless force–strain relationship proposed by Zajac (1989) for the tendon and using values of maximum isometric force and tendon slack length taken from the literature (Delp 1990). The properties of the muscles and ligaments included in the developed femur model are made available in the electronic supplementary material. Artificial joint structures composed of a layer of cortical bone, a layer of cartilage-like material, and truss elements connected to the joint centre or axis were defined at the hip, tibiofemoral and patellofemoral joints (Fig. 1c–e, respectively) to allow for the transfer of forces to the femur. The tensor fascia latae was defined in a similar way to allow the muscle to wrap around the greater trochanter (Fig. 1c). The surfaces between the femur and the joint structures were tied.

An artificial pelvic structure (Fig. 1c, in red) connected the acetabular region to muscle insertion points on the pelvis, sacrum, lumbar spine and a point representative of L5S1, as proposed by Phillips (2009). The structure was connected to and was allowed to rotate about the centre of the hip joint structure. Intersegmental forces and moments calculated as detailed in Sect. 2.2 were applied at the connection point, coincident with the centre of the femoral head. To aid stability of the pelvic structure, the L5S1 point was connected to ground via a spring element with a stiffness of 10 N/mm in the anterior–posterior direction and negligible stiffness in the other two directions. A functional axis about which knee flexion occurred was defined (Fig. 1, blue dashed line) by a beam element connecting two points on the tibiofemoral joint structure (Fig. 1d). The medial point was fixed against displacement, allowing for the femur to pivot about the medial condyle on the tibial plateau (Johal et al. 2005). Fixed constraints were applied at the insertion points of muscles and ligaments on the tibia and fibula, and segment positions were updated according to the kinematics of the two activities. The resulting model with all the joints, muscles and ligamentous structures is shown in Fig. 1.

### Multiple load cases

Two daily activities were modelled: walking and stair climbing. These were selected as they produce the highest hip joint contact forces (JCFs) (Bergmann et al. 2001) amongst the most frequent recorded activities of daily living (Morlock et al. 2001). Using gait data available in the HIP98 database (Bergmann et al. 2001) as input into a unilateral musculoskeletal model of the lower limb (Modenese and Phillips 2012; Modenese et al. 2011), the joint angles, and intersegmental moments and forces for the investigated activities performed by patient HSR (Bergmann et al. 2001) were calculated through the inverse kinematics and inverse dynamics tools available in OpenSim (Delp et al. 2007). In the finite element simulations, 40 equally spaced frames were considered for walking (trial HSRNW4) and stair climbing (trial HSRSU6), resulting in eighty load cases, including the frame where the peak hip joint force was recorded (Bergmann et al. 2001). The joint angles were used to update the initial position of the pelvic region (black reference system, Fig. 1b), the femur (red reference system, Fig. 1b) and the tibial region (blue axis, Fig. 1b) for each frame, while the intersegmental joint forces and moments calculated by the musculoskeletal model were applied at the hip joint.

### Bone adaptation algorithm

#### Identification of target material orientations from the guiding frame

**Fig. 2:**
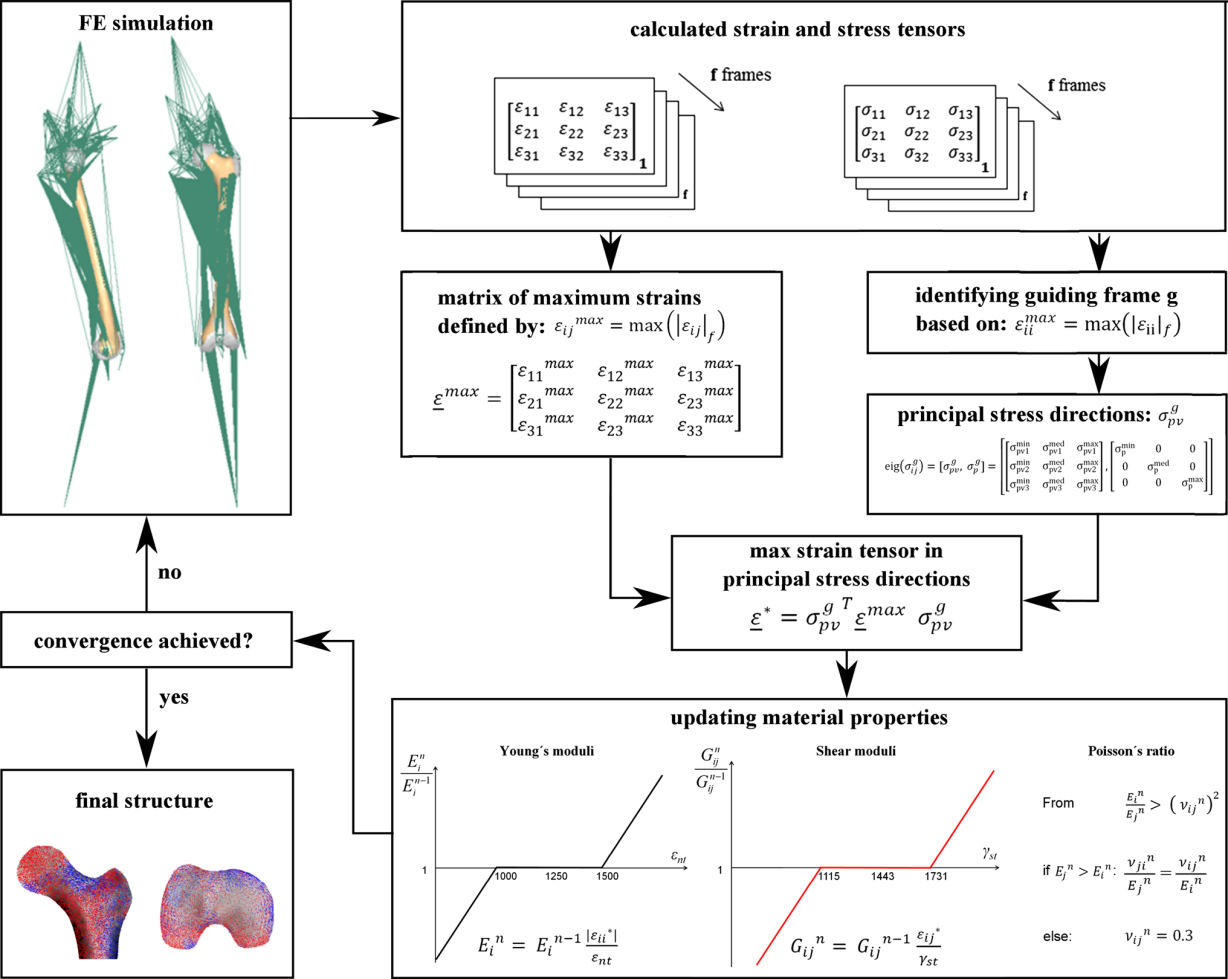
Key steps in updating the orthotropic material properties and directionality for the multiple load case adaptation process

In the single load case orthotropic adaptation algorithm previously introduced (Geraldes and Phillips 2014), each element’s material orientations were rotated in order to match the local principal stress directions in agreement with *Wolff’s Law *(Cowin 1986). This algorithm was modified to include multiple load cases (Fig. 2). The maximum strain components across all frames from the daily activities were selected for each element in order to produce a strain field envelope containing the maximum driving stimuli for the material properties and orientations. This stimulus envelope was used instead of combining multiple load cases into a single load case since, from a structural engineering perspective, bone is assumed to be adapted to adequately resist all non-traumatic loads that it is subjected to.

All the load cases from both activities were analysed in parallel, and the strain and stress tensors extracted for every element for every frame. The guiding load frame, *g*, for the adaptation process in each element was selected as the one where the maximum absolute normal strain value, $\varepsilon _{n}^\mathrm{max}$, could be found (Eq. 1), across $f=1,\ldots ,80$ frames.

1 $$\begin{aligned} \varepsilon _{n}^\mathrm{max} =\hbox {max}\left( {\left| {\varepsilon _{{ii}} } \right| _f } \right) , \quad i=1,2,3 \end{aligned}$$

After selecting the guiding load frame for each of the elements in the femoral mesh, the guiding stress tensor, $\sigma _{ij}^g$, was defined as the stress tensor associated with that frame (Eq. 2).

2 $$\begin{aligned} \sigma _{ij}^g =\left[ {{\begin{array}{lll} {\sigma _{11}{}^{g}}&{}\quad {\sigma _{12}{} ^{g}}&{}\quad {\sigma _{13}{}^{g}} \\ {\sigma _{21}{}^{g}}&{}\quad {\sigma _{22}{}^{g}}&{} \quad {\sigma _{23}{}^{g}} \\ {\sigma _{31}{}^{g}}&{}\quad {\sigma _{23}{}^{g}}&{} \quad {\sigma _{33}{}^{g}} \\ \end{array} }} \right] \end{aligned}$$

Similar to the single load case algorithm (Geraldes and Phillips 2014), the principal stresses, $\sigma _\mathrm{p}^{g}$, and orientations, $\sigma _\mathrm{pv}^g$, for each element were found by performing an eigenanalysis of the guiding stress tensor (Eq. 3).

3 $$\begin{aligned}&\hbox {eig}\left( {\sigma _{ij}^g } \right) =\hbox {[}\sigma _\mathrm{pv}^g \hbox {,}\sigma _\mathrm{p}^g\hbox {]}\nonumber \\&\quad =\left[ {\left[ {{\begin{array}{lll} {\sigma _{\mathrm{pv1}}^{\mathrm{min}} }&{}\quad {\sigma _{\mathrm{pv1}}^{\mathrm{med}} }&{}\quad {\sigma _{\mathrm{pv1}}^{\mathrm{max}} } \\ {\sigma _{\mathrm{pv2}}^{\mathrm{min}} }&{}\quad {\sigma _{\mathrm{pv2}}^{\mathrm{med}} }&{}\quad {\sigma _{\mathrm{pv2}}^{\mathrm{max}} } \\ {\sigma _{\mathrm{pv3}}^{\mathrm{min}} }&{}\quad {\sigma _{\mathrm{pv3}}^{\mathrm{med}} }&{}\quad {\sigma _{\mathrm{pv3}}^{\mathrm{max}} } \\ \end{array} }} \right] ,\left[ {{\begin{array}{lll} {\sigma _\mathrm{p}^{\mathrm{min}} }&{}\quad 0&{}\quad 0 \\ 0&{}\quad {\sigma _\mathrm{p}^{\mathrm{med}} }&{}\quad 0 \\ 0&{}\quad 0&{}\quad {\sigma _\mathrm{p}^{\mathrm{max}} } \\ \end{array} }} \right] } \right] \nonumber \\ \end{aligned}$$

In each iteration, the axes defining the orthotropic orientations were aligned to the vectors defining the directions of the principal stresses. In order to assess the influence of the multiple load case envelope on the orthotropic material property and orientation distribution against the commonly used single load case approach, the adaptation algorithm was also run for a single frame of the HSRNW4 trial associated with the peak recorded hip JCF for walking (Bergmann et al. 2001), similar to bone remodelling studies where only a single load case was considered.

#### Selection of target stimulus

For each element, the maximum absolute values, $\varepsilon _{ij} {}^\mathrm{max}$, for each of the strain components, $\varepsilon _{ij}$, were chosen from across all frames, $f=1,\ldots ,80$ (Eq. 4).

4 $$\begin{aligned} \varepsilon _{ij}{}^\mathrm{max}=\max \left( {\left| {\varepsilon _{ij} } \right| _f } \right) , \quad i=1,2,3,\,\, j=1,2,3 \end{aligned}$$

The maximum strain matrix, $\underline{\varepsilon }^\mathrm{max}$, is then defined as (Eq. 5):

5 $$\begin{aligned} \underline{\varepsilon }^\mathrm{max}=\left[ {{\begin{array}{lll} {\varepsilon _{11}{}^\mathrm{max}}&{}\quad {\varepsilon _{12}{}^\mathrm{max}}&{}\quad {\varepsilon _{13}{}^\mathrm{max}} \\ {\varepsilon _{21}{}^\mathrm{max}}&{}\quad {\varepsilon _{22}{}^\mathrm{max}}&{}\quad {\varepsilon _{23}{}^\mathrm{max}} \\ {\varepsilon _{31}{}^\mathrm{max}}&{}\quad {\varepsilon _{23}{}^\mathrm{max}}&{}\quad {\varepsilon _{33}{}^\mathrm{max}} \\ \end{array} }} \right] \end{aligned}$$

The strain stimulus matrix, $\underline{\varepsilon }^{*}$, associated with the directions of the principal stresses from the guiding frame, $\sigma _\mathrm{pv}^g$, was used as the driving stimulus of the adaptation process (Eq. 6).

6 $$\begin{aligned} \underline{\varepsilon }^{{*}}={\sigma _\mathrm{pv}^g} ^{T}\underline{\varepsilon }^\mathrm{max} \sigma _\mathrm{pv}^g \end{aligned}$$

Orthotropic bone adaptation progressed using $\underline{\varepsilon }^{*}$ as the driving stimulus for the material properties of each element and $\sigma _\mathrm{pv}^g$ to align the orthotropic orientation, in agreement with the *Mechanostat *(Frost 1987) and trajectory (Skedros and Baucom 2007) hypotheses. $\underline{\varepsilon }^{*}$ and $\underline{\varepsilon }^\mathrm{max}$ were observed to converge during the first five iterations.

#### Material properties adaptation

The material properties were adjusted in order to bring the local strains within the remodelling plateau defined around a normal target strain, $\varepsilon _\mathrm{nt}$, of 1250  $\mu$strain with a margin of $\pm$0.2$\varepsilon _\mathrm{nt}$, according to previous work by the authors (Geraldes and Phillips 2014; Phillips et al. 2015). In each iteration *n*, the orthotropic Young’s moduli, $E_i{}^{n}$, of elements with normal strains outside the remodelling plateau were updated proportionally to the absolute value of the associated normal local strain stimulus, $\varepsilon _{ii}{}^{*}$ (Eq. 7), limited between 10 MPa and 30 GPa (Geraldes and Phillips 2014).

7 $$\begin{aligned} E_i{}^{n}=E_i{}^{n-1}\frac{\left| {\varepsilon _{ii} ^{\mathrm{*}}} \right| }{\varepsilon _\mathrm{nt} } \end{aligned}$$

Poisson’s ratios for each element, $\nu _{ij}{}^{n},$ were assumed to be less than or equal to 0.3 and altered so that the compliance matrix remained always positive definite, in order to satisfy the thermodynamic restrictions on the elastic constants of bone (Cowin and Buskirk 1986; Eq. 8).

8 $$\begin{aligned} \frac{E_i{}^{n}}{E_j{}^{n}}>\left( {\nu _{ij}{}^{n}} \right) ^{2} \end{aligned}$$

If $\hbox {E}_i{}^{n}$ was greater than $\hbox {E}_j{}^{n},\nu _{ij}{}^{n}$ was kept at 0.3 while $\nu _{ji}{}^{n}$ was adjusted such that the following equality constraint was maintained (Eq. 9).

9 $$\begin{aligned} \frac{\nu _{ji}{}^{n}}{E_{j}{}^{n}}=\frac{\nu _{ij}{}^{n}}{E_{i}^{n}} \end{aligned}$$

Similar to the normal strain adaptation process, a target shear strain, ${\varepsilon }_{\mathrm{st}}$, was required to drive adaptation of the shear moduli and was calculated as explained below. Octahedral shear strain, $\gamma _o$, is defined according to Eq. 10.

10 $$\begin{aligned} \gamma _o =\frac{2}{3}\sqrt{\left( {\varepsilon _{11}-\varepsilon _{22} } \right) ^{2}+\left( {\varepsilon _{22} -\varepsilon _{33} } \right) ^{2}+\left( {\varepsilon _{33} -\varepsilon _{11} } \right) ^{2}+6\left( {\varepsilon _{12} ^{2}+\varepsilon _{23} ^{2}+\varepsilon _{13} ^{2}} \right) }\nonumber \\ \end{aligned}$$

This strain invariant has been used as a tissue adaptation stimulus (Carter et al. 1998; Shefelbine et al. 2005), and it is assumed that corresponding target values of normal and shear strain would result in the same value of the strain invariant. For a pure axial deformation (${\varepsilon }_{22} ={\varepsilon }_{33} ={\varepsilon }_{12} ={\varepsilon }_{13} ={\varepsilon }_{23} =0;{\varepsilon }_{11} ={\varepsilon }_{\mathrm{nt}} )$, octahedral shear strain is found (Eq. 11):

11 $$\begin{aligned} \gamma _o =\left( {\frac{2\sqrt{2}}{3}} \right) \varepsilon _\mathrm{nt} \end{aligned}$$

Similarly, for a pure shear deformation (${\varepsilon }_{11} ={\varepsilon }_{22} ={\varepsilon }_{33} ={\varepsilon }_{13} ={\varepsilon }_{23} =0,{\varepsilon }_{12} ={\varepsilon }_{\mathrm{st}} )$, octahedral shear strain is found (Eq. 12).

12 $$\begin{aligned} \gamma _o =\left( {\frac{2\sqrt{6}}{3}} \right) \varepsilon _{\mathrm{st}} \end{aligned}$$

Equations 11 and  12 allow a target engineering shear strain, $\gamma _\mathrm{st} ,$ to be defined (Eq. 13).

13 $$\begin{aligned} \gamma _\mathrm{st} =2\varepsilon _\mathrm{st} =2\left( {\frac{\sqrt{2}}{\sqrt{6}}} \right) \varepsilon _\mathrm{nt} =1443\, \mu \hbox {strain} \end{aligned}$$

It should be noted that octahedral shear strain was not used to drive the adaptation process, but simply as a method to establish a target shear strain. The shear moduli of each element, $G_{ij}{}^{n}$, were updated proportional to $\gamma _\mathrm{st}$ with a plateau of $\pm 0.2\gamma _\mathrm{st}$, following the same method used for the Young’s moduli (Eq. 14) and were limited between 5 MPa and 15 GPa.

14 $$\begin{aligned} G_{ij}{}^{n}=G_{ij}{}^{n-1}{\frac{\left| \varepsilon _{ij}\right| {}^{\mathrm{*}}}{\varepsilon _\mathrm{st} }}, \quad \hbox { where }i\ne j \end{aligned}$$

A state of convergence was considered to have been achieved when the average change in Young’s moduli of all elements with maximum absolute normal strain values above 250 $\mu$strain and Young’s moduli above 100 MPa was less than 2 % between successive iterations.

The resulting material properties and directionalities were compared with CT and micro-CT ($\mu$CT) scans of femoral specimens. Details of the bone density calculations and the imaging data are available in the electronic supplementary material. Sensitivity studies were also performed to assess the dependency of the predicted results with mesh properties and starting configuration of material properties for 2D and 3D and have been described in Geraldes (2013). Comparison between the two models was not affected by either.

## Results

**Fig. 3:**
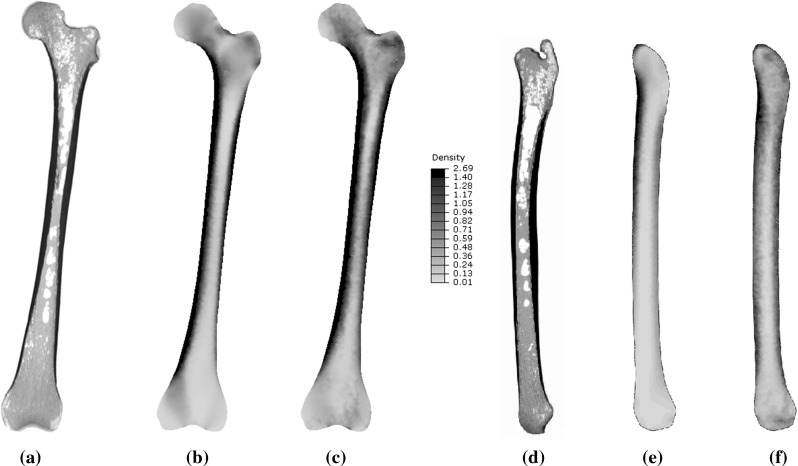
Coronal and sagittal slices of the density distributions $(\hbox {g/cm}^{3})$ for **a, d** a CT scan of the whole femur, converged models for **b, e** a single load case and **c, f** multiple load cases

Convergence of the model subjected to a single load case and multiple load cases was achieved after 29 and 30 iterations respectively. Figure 3 shows a coronal slice of a CT scan of the whole femur (a, d) compared with the predicted density distribution for the orthotropic model based on a single load case (b, e) and multiple load cases (c, f). All elements with a density above $1.4\,\hbox {g/cm}^{3}$ were grouped together as dense cortical bone, in order to allow for better visualisation of the predicted density distributions for the trabecular bone. The mean, 5th, 25th, 50th, 75th and 95th percentile of element values of Young’s and shear moduli and density for both single and multiple load case models are shown in Table 1. The converged multiple load case model resulted in 95 % larger average representative Young’s modulus, $\hbox {E}_\mathrm{rep}$, and 59 % larger average density, $\rho$, across the model than the single load case orthotropic model, as calculated according to equations S1 to S4, available in the electronic supplementary material. An alternative approach to the use of an empirical relationship was also considered in deriving bone density from the orthotropic elastic constants and is included in the electronic supplementary material.

**Table 1:** Mean, maximum, and 5th, 25th, 50th, 75th and 95th percentile of element values for $E_\mathrm{mean} , G_\mathrm{mean} , E_i ,G_{ij}$ for the converged single and multiple load case models

Percentile of elements	$\rho _\mathrm{mean} (\hbox {g/cm}^{3})$	$\hbox {E}_\mathrm{mean }$(MPa)	$\hbox {G}_\mathrm{mean }$(MPa)	$\hbox {E}_{1 }$(MPa)	$\hbox {E}_{2 }$(MPa)	$\hbox {E}_{3}$(MPa)	$\hbox {G}_{12 }$(MPa)	$\hbox {G}_{13 }$(MPa)	$\hbox {G}_{23 }$(MPa)
Single load case									
5th	0.034	44	5	38	10	28	5	5	5
25th	0.147	396	5	474	86	229	5	5	5
50th	0.313	1217	5	1510	289	917	5	5	5
75th	0.689	3933	5	3921	900	2735	5	5	5
95th	1.44	11,800	5	23,825	2700	15,696	5	5	5
Mean	0.456	2689	5	4465	610	2992	5	5	5
Multiple load case									
5th	0.039	54	5	51	13	27	5	5	5
25th	0.178	524	5	593	119	322	5	5	5
50th	0.522	2600	5	2700	594	1973	5	5	5
75th	1.440	11,800	11	14,327	1875	4303	5	6	5
95th	1.494	12,429	811	30,000	2689	30,000	60	1790	13
Mean	0.723	5245	135	8765	1262	5708	75	298	33

**Fig. 4:**
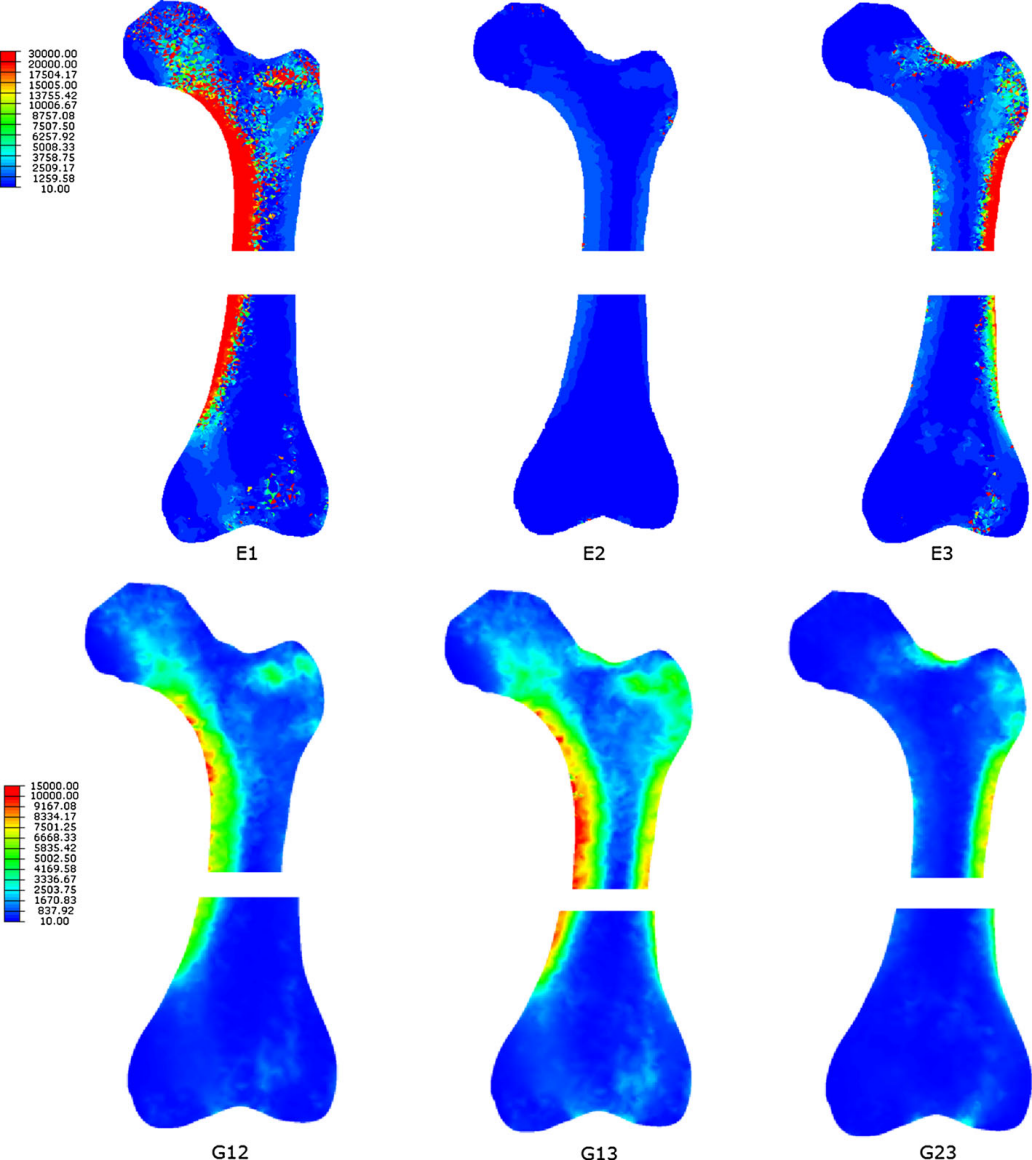
Coronal distributions of $\hbox {E}_{1}$ (*top*, *left*), $\hbox {E}_{2}$ (*top*, *middle*) and $\hbox {E}_{3}$ (*top*, *right*) and G$_{12}$ (*bottom*, *left*), G$_{13}$ (*bottom*, *middle*) and G$_{23}$ (*bottom*, *right*) in MPa for the proximal and distal regions of the multiple load case femur model

Coronal sections of the converged Young’s moduli are represented in Fig. 4 for the proximal and distal regions of the multiple load case femur model: $\hbox {E}_{1}$ (left), $\hbox {E}_{2}$ (middle) and $\hbox {E}_{3}$ (right). Young’s modulus values were higher for $\hbox {E}_{1}$ and $\hbox {E}_{3}$ than for $\hbox {E}_{2}$. $\hbox {E}_{1}$ was generally associated with compression and $\hbox {E}_{3}$ with tension. The converged shear moduli for the same regions of the femur: $\hbox {G}_{12}$ (left), $\hbox {G}_{13}$ (middle) and $\hbox {G}_{23}$ (right), are depicted in Fig. 4 (bottom). Shear modulus values were higher for $\hbox {G}_{13}$ than for $\hbox {G}_{12 }$ and $\hbox {G}_{23}$.

**Fig. 5:**
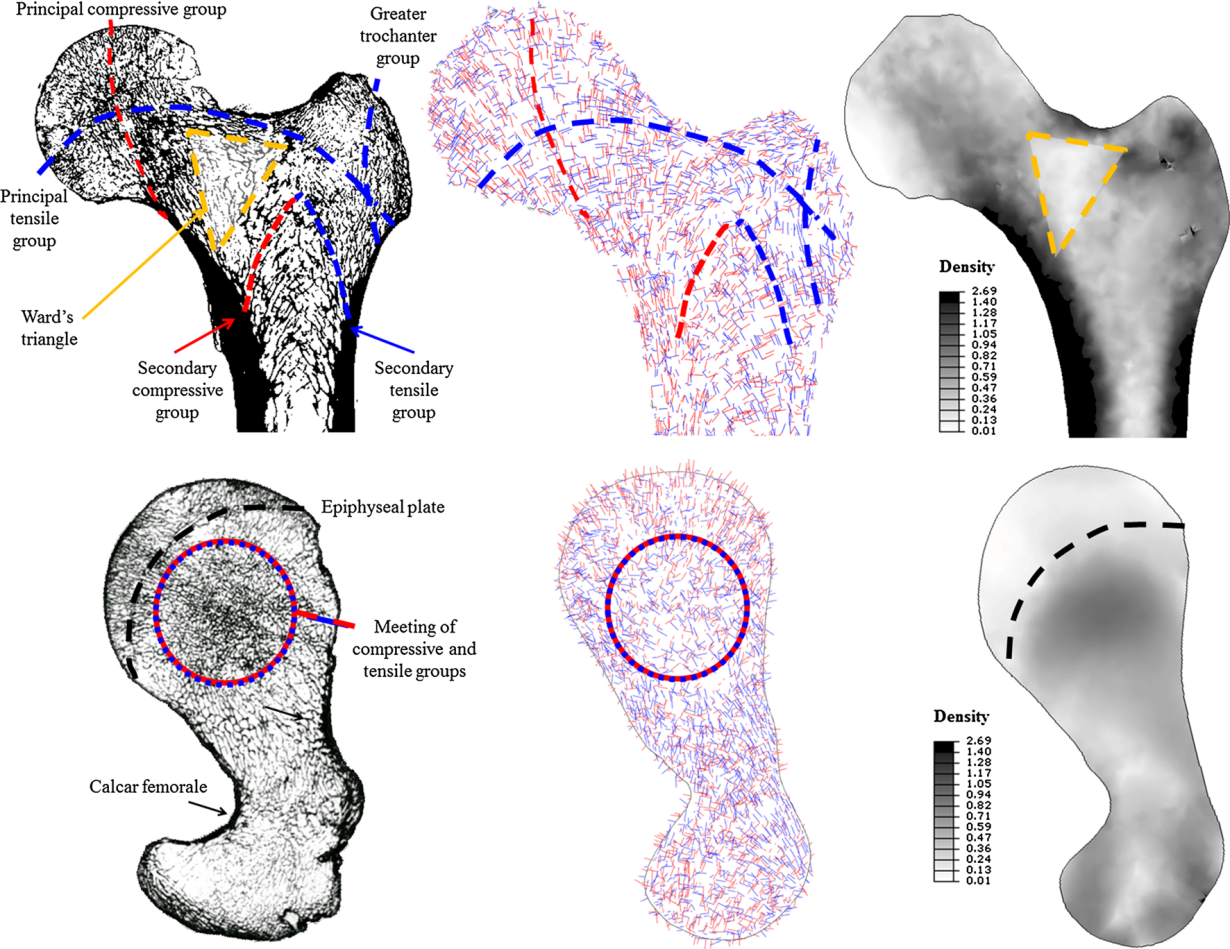
Predicted density (*right*, in $\hbox {g/cm}^{3})$ and dominant material orientations (*middle*) for a coronal (*top*) and transverse (*bottom*) section of the converged proximal femur undergoing multiple load cases. Legends highlighting the most interesting features identified by Singh et al. (1970) (*top*, *left*) and Tobin (1955) (*bottom*, *left*) were superimposed onto a $\mu$CT slice of the same region. The material orientations associated with $\hbox {E}_{1}$ are shown in *red* and $\hbox {E}_{3}$ in *blue*

**Fig. 6:**
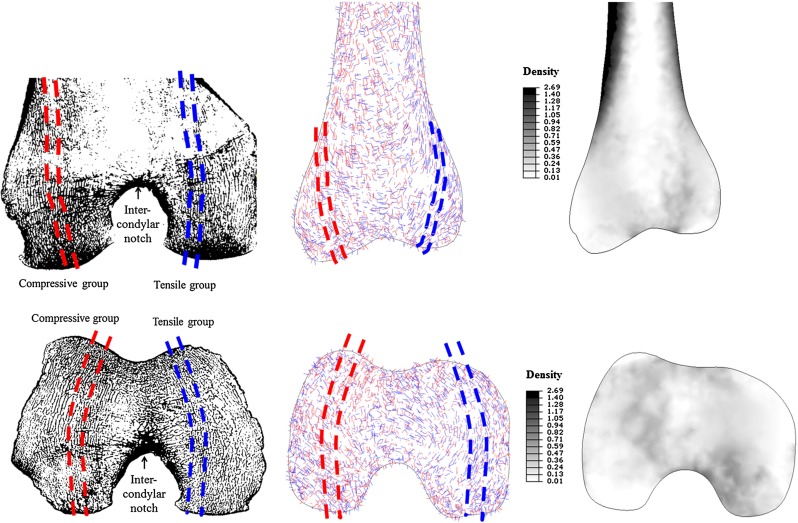
Predicted density (*right*, in g/cm$$^{3})$$ and dominant material orientations (*middle*) for a coronal (*top*) and transverse (*bottom*) section of the converged distal femur undergoing multiple load cases. Legends highlighting the most interesting features identified by Takechi (1977) were superimposed onto a $$\mu $$CT slice of the same region. The material orientations associated with $$\hbox {E}_{1}$$ are shown in *red* and $$\hbox {E}_{3}$$ in *blue*

In Figs. 5 and 6, the predicted density (right) and dominant material orientations (middle) for coronal and transverse sections are shown, respectively, of the proximal (Fig. 5) and distal (Fig. 6) femur, compared to $\mu$CT slices for the same regions (left). The dominant material directions were defined as the orientation associated with the highest directional Young’s modulus for each element. The material orientations associated with $\hbox {E}_{1}$ are shown in red and with $\hbox {E}_{3}$ in blue.

**Fig. 7:**
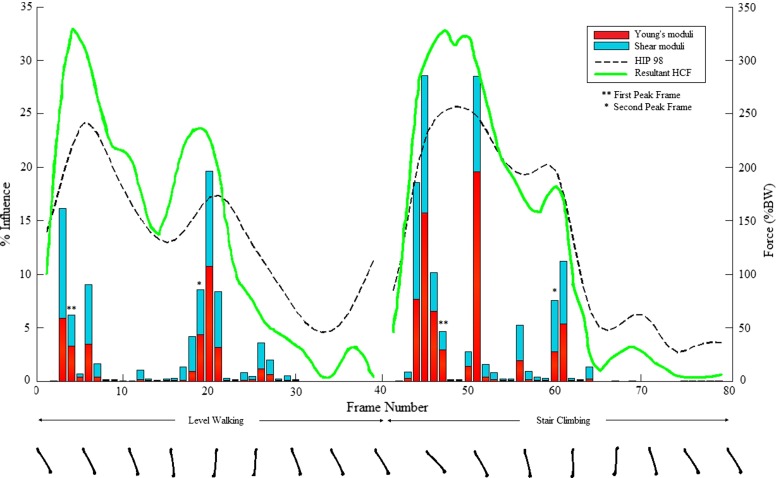
Percentage of elements influenced by each load case (frames 1–40 for walking and 41–80 for stair climbing) for Young’s moduli (*red*) and shear moduli (*blue*) adaptation. The hip JCFs (%BW) calculated by the proposed model (*solid green line*) and measured by instrumented prosthesis (HIP98, *dashed black line*) are also shown. The frames where the first peak (**) and second peak (*) occurred are *highlighted*. The orientation of the femur, highlighting its flexion angle in the sagittal plane, are included at the bottom

Figure 7 shows the plot of the percentage of elements for which each frame is the guiding frame with respect to the Young’s moduli (red) and shear moduli adaptation (blue), as well as a representation of the femur’s orientation in the sagittal plane (bottom), in order to ease spatial visualisation. The predicted hip JCFs [% body weight (BW)] for the model (green dashed line) were higher than those measured and reported in the HIP98 data set (black dashed line; Bergmann et al. 2001). Pearson’s $\rho$ and root-mean-squared error (RMSE, in %BW) between the predicted and the measured resultant hip contact forces for walking and stair climbing are shown in Table 2.

**Fig. 8:**
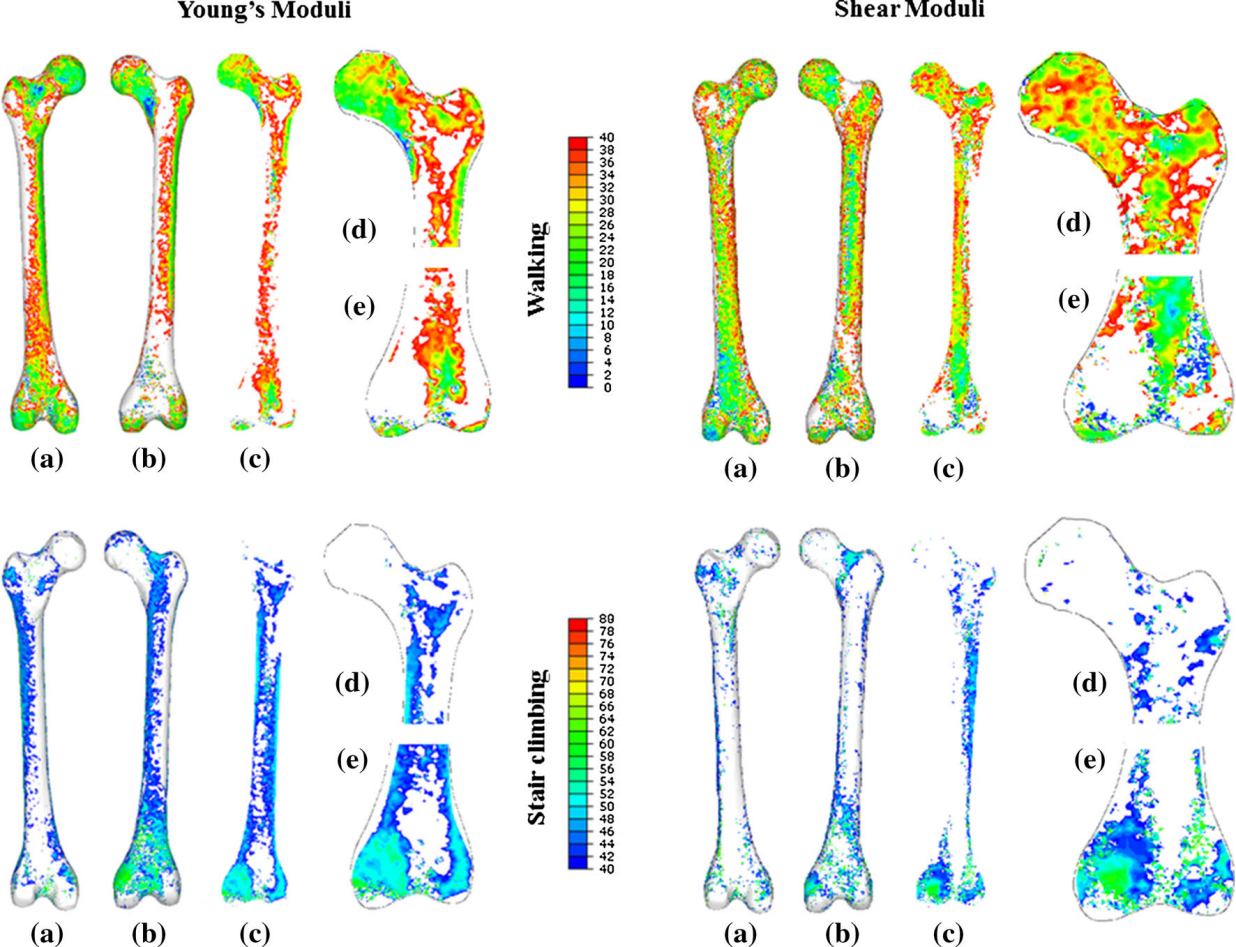
Guiding load frames indices for Young’s moduli (*left*) and shear moduli (*right*) for walking (*top*) and for stair climbing (*bottom*): posterior (**a**), anterior (**b**) and coronal section (**c**) views of the whole femur and coronal section views of its proximal (**d**) and distal (**e**) regions

The dominant frame indices for each element are displayed for posterior and anterior views of the whole femur, as well as a coronal section of the whole length, proximal and distal regions for the adaptation process of Young’s moduli (Fig. 8, left) and shear moduli (Fig. 8, right). The indices are split into the two activities modelled: walking (top) and stair climbing (bottom), making evident that the most influential phases of the gait cycle for bone adaptation are early stance during stair climbing and the entire stance phase for walking.

## Discussion

The cortical thickness along the lateral aspect of the femoral shaft and trabecular density in the distal regions are a better match with the femoral CT slice when multiple load cases are considered compared to the single load case (Fig. 3). Quantitative analysis of the predicted spatial density distributions and CT images are included in the electronic supplementary material. Regions where non-orthogonal trabecular crossing was expected (such as the area where the tensile and compressive trabecular groups meet in the femoral head and in the intertrochanteric region, as suggested in the trajectorial hypothesis proposed by Skedros and Baucom (2007)) coincided with regions of higher values of predicted shear moduli, in agreement with the first hypothesis of the study (Fig. 4, bottom). Table 1 highlights that shear resistance does not affect bone adaptation under a single load case as low values of shear modulus are produced. However, when multiple load cases representing common daily living activities are applied, shear adaptation needs to be considered as 25 % of the elements present increased shear resistance.

For the model subjected to multiple load cases, all main trabecular groups and Ward’s triangle (Singh et al. 1970) were predicted for the proximal femur (Fig. 5, top). The arrangement of the orthotropic axes is consistent with observations of trabeculae running perpendicular to the articular surface and the calcar femorale (Fig. 5, bottom). The coronal and transverse sections also show three typical features: the meeting of the secondary compressive group and the tensile greater trochanter group at the apex of the intertrochanteric arch; the meeting of the principal tensile and compressive groups in the centre of the femoral head; and a crescent-shaped region of density transition near the epiphyseal plate (Tobin 1955).

The vertical alignment of the tensile and compressive trabecular groups parallel to the bone axis and the surface contour of the condyles, in order to transfer the loads arising from the daily activities through the knee joint (Takechi 1977), is correctly predicted (Fig. 6, top), as well as the trabecular arrangements in a transverse section of the condyles (Takechi 1977; Fig. 6, bottom). The inclusion of multiple load cases allowed for realistic predictions of the trabecular structure directionality at a continuum level for the whole femur, and it is suggested that the modelling of more extreme activities where higher flexion angles, quadriceps muscles activations and patellofemoral forces are involved (such as sit to stand) could further improve the comparison in the distal region.

The high Pearson’s coefficients showed a strong correlation between the predicted hip JCFs and the forces reported in HIP98 for the same activities (Fig. 7; Table 2). We conclude that the proposed model is capable of producing a load environment in the femur close to the in vivo situation, an important consideration in biomechanical investigations (Erdemir et al. 2012). The impact of different frames on Young’s moduli adaptation is clearly observed in Fig. 7 (in red), with 7 frames producing the stimulus necessary to influence more than 5 % of the elements each. The frames where the peak predicted hip JCF occurred are not amongst the top five dominant frames. These correspond to either an almost vertical positioning of the femur or high flexion angles, which may be associated with higher muscle forces, particularly in the quadriceps when flexion occurs. The influence of the different load cases on shear moduli adaptation can also be observed in Fig. 7 (blue). More frames influence shear moduli than Young’s moduli, and some overlap in importance. This is expected, since the load cases that produce high axial strains in the cortical shaft will also generate high torsional moments in the femoral neck and the cortical shaft (Garden 1961; Tobin 1955; Varghese et al. 2011) resulting in high shear.

The contribution of both daily activities to the converged material properties in vast, independent regions of the femur is clear (Figs. 7, 8), in agreement with findings from a mesoscale structural approach to bone adaptation (Phillips et al. 2015) and with the second hypothesis of this study. Walking is shown to influence the Young’s moduli in regions of the femoral head, inferior part of the femoral neck, greater trochanter, posterior-lateral aspects of the femoral shaft and posterior aspect of the condylar region (Fig. 8, left). Stair climbing dominates in the regions of the superior part of the femoral neck, lesser trochanter, medial aspect of the cortical shaft and anterior aspect of the distal femur. Early stance is more influential in stair climbing than walking for bone adaptation. The material properties of the femoral shaft and the femoral neck are visibly influenced by both activities, with simple load case models demonstrated to underestimate the average stiffness and density of the femur. Bone adaptation studies need to consider the possibility that distinct bone regions could experience maximum loading in frames of the considered activities for which the JCFs are not at their peak.

**Table 2:** Pearson’s $\rho$ and RMSE (%BW) between the predicted and the measured resultant hip JCFs measured by Bergmann et al. (2001) for walking and stair climbing

Walking	
Pearson’s $\rho \;(p<0.0001)$	0.9230
RMSE (%BW)	58.08
Stair climbing	
Pearson’s $\rho \;(p<0.0001)$	0.9765
RMSE (%BW)	43.01

When looking at shear modulus adaptation (Fig. 8, right) it is evident that it is primarily driven by walking, with stair climbing load frames losing their topological dominance (Fig. 8, right). Nevertheless, the anterior aspect of the distal region and the superior anterior region of the femoral neck are dominated by stair climbing. This suggests that stair climbing could improve bone’s stiffness and resistance to normal and shear strains in these regions, potentially reducing the risk of fracture, in agreement with observations on the influence of the same activities on bone density (Allison et al. 2015; Blain et al. 2009; Jamsa et al. 2006).

Several limitations of the model need to be considered. The geometrical definition of certain muscles, such as the iliopsoas, through straight lines can result in non-physiological lines of action and moment arms compared to more detailed representation obtained in musculoskeletal models using viapoints and wrapping surfaces (Modenese et al. 2011; van Arkel et al. 2013). This geometrical limitation, together with the femur geometry used in this study, which is not personalised for subject HSR (Bergmann et al. 2001), but extracted from the muscle standardised femur (Viceconti et al. 2003), could have contributed to overprediction of the hip contact forces (Modenese et al. 2013). Intersegmental forces and moments were not applied at the tibiofemoral joint. Their omission will have influenced the predicted peak tibiofemoral forces (179 %BW and 165 %BW for walking and stair climbing, respectively), found to be lower than those measured using instrumented knee prostheses (217 %BW for walking and 250 %BW for stair climbing; D’Lima et al. 2006). It is likely that the prediction of material properties in the region was also affected by this simplification.

A relationship between number of loading cycles and target strain is required for the maintenance of bone (Ozcivici et al. 2010). High-repetition activities generating low bone strains could have similar effects to low-repetition high-impact activities generating high strains (Fehling et al. 1995; Judex and Zernicke 2000). As the lazy zone represents a $\pm$20% interval, we assigned an equal weight of 1 to every frame and activity modelled in the multiple load case model as the adaptation process will be relatively insensitive to small changes in the target strain based on the number of activity cycles, for the two chosen activities, level walking (around 10,000 cycles/day) and stair climbing (around 100 cycles/day) (Morlock et al. 2001), which have similar effects on bone adaptation in the presented model. A limitation of the proposed multiple load case scenario is that it did not include any impact activity, as the publicly available database (Bergmann et al. 2001) used in validation of the musculoskeletal model does not include impact activities such as running. Predictions may be improved by including impact activities in future work. The optimised orthotropic structure produced is independent of the duration and frequency of loading since, much like the trabecular structure that has been extensively studied in the proximal femur (Garden 1961; Singh et al. 1970; Skedros and Baucom 2007; Takechi 1977), it is the configuration that best maintains the bone strains within the threshold proposed by Frost (1987) for the envelope of load cases applied. Therefore, time- or frequency-dependent adaptation is not considered in this analysis and is acknowledged as a limitation of the proposed method.

Figures 7 and 8 show that multiple representative frames of different activities need to be included in order to reliably capture the complex in vivo mechanical environment which drives the adaptation process, a crucial limitation of previous bone adaptation studies that have only considered reduced number of frames for the activities modelled. Other mechanistic models also report physiological distributions of mass and principal directions under non-isotropic conditions and using averaged stimulus that weight each stress state. Tsubota et al. (2009) show impressive results from a voxel-based approach applied to a 3D proximal femur. Homogenisation methods have reported physiological 3D distributions and orientations for a section of the proximal femur (Bagge 2000; Fernandes et al. 1999). However, the difficulty in accurately modelling the 3D mechanical environment for the whole femur leads to these different methods being applied with simplified loading conditions and only being reported for coronal slices of the proximal femur. The use of a balanced model allows for application of the adaptation process for the complete femur, without artefacts induced by non-physiological boundary conditions. This is further explored in the electronic supplementary material.

The final product of this investigation is a heterogeneous orthotropic model of the femur informed by a physiological representation of the loading environment. The generation and development of these models can benefit research areas where prediction of local bone material properties is of key importance, with the potential to provide recommendations on which physical activities most beneficially influence bone health. In particular, this study highlights the importance of stair climbing in influencing the properties of the fracture-prone femoral neck region, with implications for non-pharmacological fracture prevention strategies based on exercise. The proposed algorithm could be extended to other bones, and future work could look into predictions of bone adaptation around bone–implant interfaces or the influence of bone degradation caused by osteoporosis.

## Electronic supplementary material

Below is the link to the electronic supplementary material.

Supplementary material 1 (pdf 697 KB)

## Footnotes

1 doi:10.6084/m9.figshare.1419589.
