# Supplementary material for: Consideration of multiple load cases is critical in modelling orthotropic bone adaptation in the femur
Source: Biomech Model Mechanobiol. 2015 Nov 17;15(5):1029–42. doi: 10.1007/s10237-015-0740-7 (PMC5021760; doi:10.1007/s10237-015-0740-7)
Supplement: Supplementary file 1 — Supplementary material 1 (pdf 697 KB) [file 10237_2015_740_MOESM1_ESM.pdf]

Consideration of multiple load cases is critical in modelling orthotropic bone adaptation in the femur

Diogo M. Geraldes [1, 2], Luca Modenese [1, 3, 4] and Andrew T.M. Phillips [1]

[1] Structural Biomechanics, Department of Civil and Environmental Engineering, Skempton Building, Imperial College London, London, UK.

[2] Biomechanics Group, Department of Mechanical Engineering City and Guilds Building, Imperial College London, London, UK.

[3] Department of Mechanical Engineering, Sir Frederick Mappin Building, Mappin Street, University of Sheffield, Sheffield, UK

[4] INSIGNEO Institute for *in silico* Medicine, The University of Sheffield, Sheffield, UK

Corresponding author: Diogo M. Geraldes [2], geraldes@imperial.ac.uk +44 (0) 207 594 1986

Electronic Supplementary Material

Manuscript published in Biomechanics and Modeling in Mechanobiology

DOI: 10.1007/s10237-015-0740-7

Properties of the muscles and ligaments

Table S1 shows the values for the properties of the muscles and ligaments included in the model: peak contractile forces (F_{peak}^{M}), tendon slack length (L_{slack}^{T}) and reference stiffness values (k_{iso}^{ML}). The stiffness values were distributed by the number of connectors defined for each group, N_c.

26 muscles and 7 ligamentous structures (Figure 1, in green) were represented as groups of spring elements, in number proportional to their insertion area. Musculotendon stiffness was calculated as in Phillips (2009) based on the dimensionless force-strain relationship proposed by Zajac (1989) for the tendon using values of maximum isometric force and tendon slack length taken from the literature (Delp 1990).

The stiffness values for the ligaments included in the model were extracted from publically available experimental measurements and validated models for the cruciate and collateral knee ligaments (Butler 1989, Li et al. 1999, Mesfar and Shirazi-Adl 2006), the patellar ligament (Stäubli et al. 1996) and the iliotibial tract (Merican and Amis 2009).

Please refer to Phillips (2009) and Geraldes and Phillips (2014) for how the stiffness value was used to prescribe the force-displacement relationship for each muscle.

Table S1 - The values for the properties of the muscles and ligaments included in the model: peak contractile forces (F^{M}_{peak}), tendon slack length (L^{T}_{slack}) and reference stiffness values (k^{ML}_{iso}). The stiffness values were distributed by the number of connectors defined for each group, N_c.

**Muscle**	F^{M}_{peak} **(N)**	L^{T}_{slack} **(mm)**	k^{MT}_{iso} **(N/mm)**	N_c
Adductor brevis	285	20	499	127
Adductor longus	430	110	137	300
Adductor magnus caudalis	220	150	51	63
Adductor magnus cranialis	880	150	205	1396
Biceps femoris long head	720	341	74	1
Biceps femoris short head	400	100	140	299
Gastrocnemius lateralis	490	385	45	55
Gastrocnemius medialis	1115	408	96	148
Gemeli	110	39	99	77
Gluteus maximus	1300	132	345	406
Gluteus medius	1365	61	783	120
Gluteus minimus	585	31	660	99
Gracilis	110	140	98	1
Iliopsoas	430	90	177	50
Pectineus	175	20	306	92
Piriformis	295	115	90	28
Psoas	370	130	100	1
Quadratus femoris	225	24	328	37
Rectus femoris	780	346	79	2
Sartorius	105	40	92	1
Semimembranosus	1030	359	100	1
Semitendinosus	330	262	44	1
Tensor fascia latae	155	425	13	1
Vastus intermedius	1235	136	318	2578
Vastus lateralus	1870	157	417	695
Vastus mediatus	1295	126	360	330
Gluteal iliotibial tendon	720	N/A	85	1
Iliotibial tract	430	N/A	97	2
Patella tendon	2500	N/A	1000	2
Anterior cruciate	N/A	N/A	200	13
Lateral collateral	N/A	N/A	100	26
Medial collateral	N/A	N/A	100	28
Posterior cruciate	N/A	N/A	200	17

Imaging Data

The results produced by the orthotropic adaptation process were compared with *ex vivo* imaging data of two femur specimens. A CT scan of an ethically obtained specimen of a male cadaveric femur, 55 years old, weight 94 kg and height 188 cm, was taken with a SOMATOM Definition AS+ scanner (Siemens AG, Munich, Germany) based in the Queen Elizabeth Hospital, Birmingham, UK. The specimen was scanned at 120 kV and 38.0 mA with an effective spatial resolution of 0.71 mm. The normalised density greyscales of a coronal slice of the whole femur were compared with the predictions for single and multiple load case orthotropic adaptations. In addition, micro-CT (µCT) data for the proximal and distal regions of a male cadaveric femur, 27 years old, weight 75 kg and height 175 cm, were also ethically obtained. The specimen was scanned using a HMXST 225 CT cone beam system with a 4MP PerkinElmer Detector (Nikon Metrology, Tring, UK) based in the Natural History Museum, London, UK, at 145 kV and 150 µA and with an effective spatial resolution of 78.7 µm.

Bone density calculation

The predicted density distributions for the orthotropic models based on a single load case and multiple load cases were plotted and compared with a CT scan slice of the complete femur. From the FE simulation results, a mean Young's modulus, E_{mean}, was calculated for each element as the average of the three orthotropic Young's moduli (Equation S1).

$$E_{\text{mean}} = \frac{E_1 + E_2 + E_3}{3} \quad \text{(S1)}$$

Simultaneously, the mean shear modulus, G_{mean}, was calculated as the average of the three orthotropic shear moduli (Equation S2).

$$G_{\text{mean}} = \frac{G_{12} + G_{13} + G_{23}}{3} \quad \text{(S2)}$$

The density for each element was calculated using a modulus–density relationship measured for trabecular bone in the femoral neck (Morgan et al. 2003) (Equation S3),

$$\rho = \left(\frac{\mathrm{E}_{\text{rep}}}{6.850}\right)^{\frac{1}{1.49}} \quad \text{(S3)}$$

where E_{rep} was taken as the maximum value between E_{mean} and the equivalent value associated with G_{mean} for each element, with ν taken as 0.3 (Equation S4), in order to account for areas where the shear modulus is predominant over the Young's modulus.

$$E_{\text{rep}} = \max(E_{\text{mean}}, 2(1 + \nu)G_{\text{mean}}) \quad \text{(S4)}$$

The conversion between orthotropic properties and density was adapted from an experimentally derived curve relating mass and elastic modulus for the femoral neck (Morgan et al. 2003). The choice of this equation is discussed in Geraldes (2013). Other studies have used similar relationships to convert the orthotropic Young's moduli to density (Miller et al. 2002).

Since shear modulus adaptation was considered, it is possible for certain elements to converge with higher values of shear moduli than those that might be expected when basing their adaptation on the directional Young's moduli. This was taken into account in the attempt to calculate a representative value of Young's modulus, E_{rep}, from which the equivalent density was calculated. The criterion presented was selected as the best representation of the element's Young's modulus after a sensitivity study involving different criteria was performed.

The use of a modulus-density relationship to produce the density plots is limiting. However, since this relationship is used for both the single and multiple load case model results, it is reasonable to believe that the relative comparison between them holds. More rigorous micromechanics approaches have been put forward by Hellmich et al. (2008) and Blanchard et al. (2013) and would allow for a more accurate calculation of the density distribution in the femur. Alternatively, an extension of the method proposed by Fernandes et al. (1999) for calculating relative bone density from a unit cell with material volume in the three orthotropic directions proportional to the directional Young's moduli could be a more physiological approach and is presented in the next section.

Alternative approach for density calculation

An alternative approach to the use of an empirical relationship was also considered in deriving bone density from the orthotropic elastic constants, using a similar method to that adopted by Fernandes et al. (1999). Considering a cube of unit volume as shown in Figure S2, the relative density (ratio of solid volume to total volume) can be expressed based on the dimensions of each of the internal cuboids representing the solid phase, assumed to be isotropic.

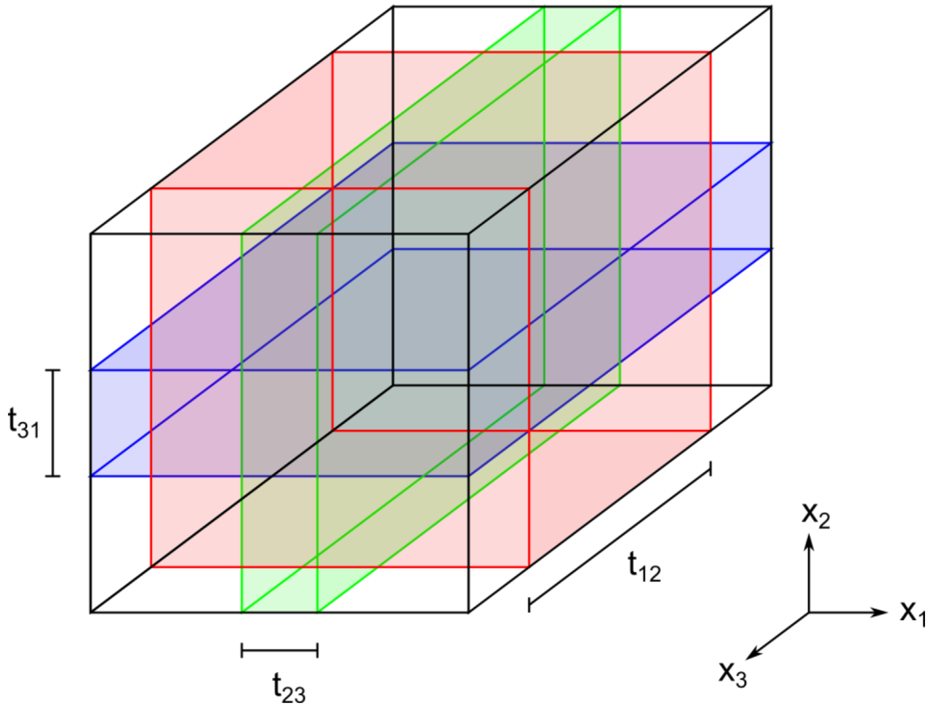

Figure S2: Cube of unit volume with internal cuboids representing the solid phase in the three orthotropic directions (x_1, x_2, x_3).

The thickness values of the cuboids can be calculated based on the ratio of the orthotropic values of Young's moduli and shear moduli compared to their maximum permissible values of 30 GPa for Young's moduli and 11.53 GPa for shear moduli, taking a Poisson's ratio of 0.3. Shear moduli values between 11.53 and 15 GPa were set to 11.53 GPa. The maximum possible thickness, t_{max} of each cuboid was limited to a value of less than 1, taking into account minimum bone porosity:

$$t_{max} = 1 - p_{min}^{\frac{1}{3}} \qquad \text{(S5)}$$

where p_{min} is the minimum bone porosity, taken as 0.02 (Cooper et al. 2007) giving a t_{max} of 0.7286. Each value of t_{ij} can be calculated as:

$$t_{ij} = \max\left(\frac{E_i}{E_{max}}, \frac{G_{ij}}{G_{max}}\right) t_{max} \qquad \text{(S6)}$$

where E_{max} and G_{max} are the maximum permissible values of Young's moduli and shear moduli, respectively. The relative density based on the elastic constants can be calculated as:

$$\bar{\rho} = t_{12} + (1 - t_{12})(t_{23} + t_{31} - t_{23}t_{31}) \qquad \text{(S7)}$$

where $\bar{\rho}$ is the relative density. In order to compare the density values against those given by the empirical formula a tissue density of 2.11g/cm^3 was used (Morgan et al. 2003). Figure S3 shows the densities calculated for each element using both approaches.

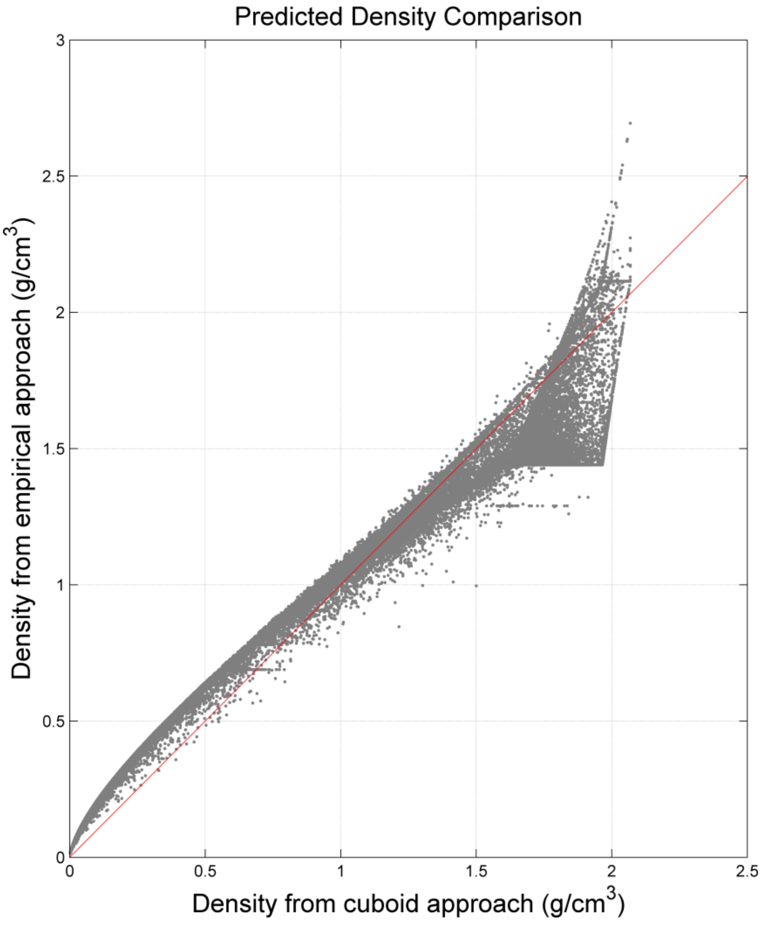

Figure S3: Comparison of the predicted densities using the cuboid and empirical approaches.

The R^2 value for a slope of 1 passing through the origin was 0.9512 with a root mean squared error (RMSE) of 0.1286. It can be seen that the cuboid approach tends to predicted lower densities in trabecular bone and higher densities in cortical bone than the adopted empirical formula. It is reasonable to conclude that while specific values of predicted density are sensitive to the chosen approach, differences in density distributions between the two loading scenarios will be similar.

Bone density comparison for the femur

A previous study by authors compared isotropic vs. orthotropic adaptation under a single load case (Geraldes and Phillips 2014) showing that the orthotropic assumption improved the prediction of bone density distribution when compared with the more commonly used isotropic approach. Inclusion of multiple load cases for a variety of frequent daily activities was suggested in order to improve the distribution of the material properties in the trabecular bone regions of the distal part of the femur and across the femoral head. Such quantitative comparison was beyond the scope of this manuscript but, for clarity purposes, we decided to include a comparison of grayscale profiles across 10 mm thick 2D slices of the proximal femur, cortical shaft and distal femur regions (Figure S4 and Table S2).

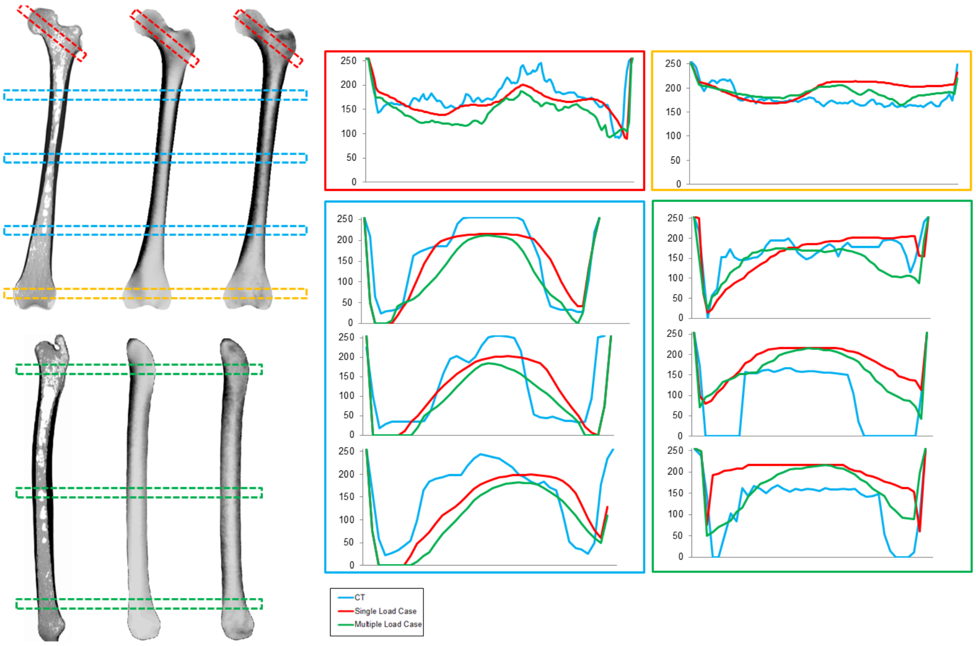

Figure S4 – Grayscale profiles for CT scan (blue), single load case model (red) and multiple load case model (green) for 10mm rectangular 2D slices across the coronal planes of the proximal femur (red box), cortical shaft regions (blue box) and distal region of the femur (orange box) and sagittal sections of the proximal, distal and cortical regions (green).

Table S2 – Pearson's product moment correlation coefficients (r, $p<0.05$) between the grayscale profiles from a CT scan, single load case model and multiple load case model. These were calculated for 10mm rectangular 2D slices the coronal planes of the proximal femur, cortical shaft regions and distal region of the femur and sagittal sections of the proximal, distal and cortical regions.

Slice	Comparison	r, p<0.05
Coronal Proximal	CT vs Single Load Case	0.68
	CT vs Multiple Load Case	0.72
Coronal Shaft 1	CT vs Single Load Case	0.85
	CT vs Multiple Load Case	0.91
Coronal Shaft 2	CT vs Single Load Case	0.70
	CT vs Multiple Load Case	0.78
Coronal Shaft 3	CT vs Single Load Case	0.67
	CT vs Multiple Load Case	0.68
Coronal Distal	CT vs Single Load Case	0.22
	CT vs Multiple Load Case	0.68
Sagittal Proximal	CT vs Single Load Case	0.72
	CT vs Multiple Load Case	0.72
Sagittal Shaft	CT vs Single Load Case	0.70
	CT vs Multiple Load Case	0.71
Sagittal Distal	CT vs Single Load Case	0.52
	CT vs Multiple Load Case	0.81

The multiple load case model produced grayscale distributions with larger Pearson's product moment correlation coefficients than the single load case model, particularly in the distal region of the femur, showing better predictions in the spatial distribution of bone density, as expected.

The importance of a physiological loading environment of the femur

It is accepted that in order to model the behaviour of bone remodelling with FE models, the driving stimulus of the adaptation process needs to be physiologically meaningful (Bitsakos et al. 2005). This involves careful selection of the loading and boundary conditions applied and has been thoroughly explored in literature. Initial models of the femur were fixed at the distal end and with the hip joint reaction force and muscle forces included as point loads (Taylor et al. 1996). Lengsfeld et al. (1996) showed that the femoral strain pattern and principal stress orientation is sensitive to the resultant joint contact forces and muscle forces at the hip joint, particularly within the coronal plane. Duda et al. (1998) further demonstrated the importance of considering a fully balanced loading configuration in order to reproduce a physiological strain distribution, rather than other loading configurations. Polgar et al. (2003) found that when muscles with large attachment areas were modelled as point loads, the stress and strain distributions were affected not only in the surroundings of the load application point but also on the internal surface of the cortical shaft. Although computationally efficient, this simplification can result in unrealistic displacement and strain values, and affects the results from elements in the FE model surrounding the point of load application. Speirs et al. (2007) showed the effect of different boundary constraints in the deflection, strain patterns magnitudes and reaction forces of the intact femur. The inclusion of non-physiological constraints, such as fully constraining a node in each condyle or the cortical shaft, led to large reaction forces, altered strain patterns in the cortex and non-physiological deflections of the femur (Phillips 2009).

It is clear from literature that boundary and loading conditions play an important role in the resulting predicted stress and strain distributions in the femur, with an increase

in their physiological significance associated with more physiological results. Therefore, the focus of this work was directed to the effect of multiple load cases in the predicted bone structure. The improvement to the adaptation results of physiological loading and boundary conditions when compared to simplified models is discussed in detail in Geraldes (2013). The authors therefore recommend the use of a balanced model to allow for the application of the adaptation process for the complete femur, without artefacts induced by non-physiological boundary conditions. This allows for reporting results for all regions of the femur in different anatomical planes, unlike other bone adaptation models. A similar approach that combined forces obtained from musculoskeletal analysis with finite element models has been used to expand a homogenization method to report density values for the complete scapula with reasonable agreement with CT scan data (Quental et al. 2014), study the accuracy of FE modelling of the scapula (Campoli et al. 2014), and a structural mesoscale model of the femur (Phillips et al. 2015). These studies further confirm the importance of careful physiological representation of joint loading and muscle forces in order to be able to report results for the complete bone geometry.

References

Bitsakos C, Kerner J, Fisher I, Amis AA (2005) The effect of muscle loading on the simulation of bone remodelling in the proximal femur. J Biomech 38(1): 133-9. doi: 10.1016/j.jbiomech.2004.03.005

Blanchard R, Dejaco A, Bongaers E, Hellmich C (2013) Intravoxel bone micromechanics for microCT-based finite element simulations. J Biomech 46(15): 2710-21. doi: 10.1016/j.jbiomech.2013.06.036

Butler DL (1989) Kappa Delta Award paper. Anterior cruciate ligament: its normal response and replacement. J Orthop Res 7(6): 910-21. doi: 10.1002/jor.1100070618

Campoli G, Bolsterlee B, van der Helm F, Weinans H, Zadpoor AA (2014) Effects of densitometry, material mapping and load estimation uncertainties on the accuracy of patient-specific finite-element models of the scapula. Journal of the Royal Society, Interface / the Royal Society 11(93): 20131146. doi: 10.1098/rsif.2013.1146

Cooper DM, Thomas CD, Clement JG, Turinsky AL, Sensen CW, Hallgrimsson B (2007) Age-dependent change in the 3D structure of cortical porosity at the human femoral midshaft. Bone 40(4): 957-65. doi: 10.1016/j.bone.2006.11.011

Delp SL (1990) Surgery simulation: A computer-graphics system to analyze and design musculoskeletal reconstructions of the lower limb. PhD, Stanford University

Duda GN, Heller M, Albinger J, Schulz O, Schneider E, Claes L (1998) Influence of muscle forces on femoral strain distribution. J Biomech 31(9): 841-6.

Fernandes P, Rodrigues H, Jacobs C (1999) A Model of Bone Adaptation Using a Global Optimisation Criterion Based on the Trajectorial Theory of Wolff. Comput Methods Biomech Biomed Engin 2(125-148.

Geraldes DM (2013) Orthotropic Modelling of the Skeletal System. Imperial College London

Geraldes DM, Phillips ATM (2014) A comparative study of orthotropic and isotropic bone adaptation in the femur. Int J Numer Method Biomed Eng 30(9): 873-89. doi: 10.1002/cnm.2633

Hellmich C, Kober C, Erdmann B (2008) Micromechanics-based conversion of CT data into anisotropic elasticity tensors, applied to FE simulations of a mandible. Ann Biomed Eng 36(1): 108-22. doi: 10.1007/s10439-007-9393-8

Lengsfeld M, Kaminsky J, Merz B, Franke RP (1996) Sensitivity of femoral strain pattern analyses to resultant and muscle forces at the hip joint. Medical Engineering & Physics 18(1): 70-78. doi: Doi 10.1016/1350-4533(95)00033-X

Li G, Gil J, Kanamori A, Woo SL (1999) A validated three-dimensional computational model of a human knee joint. J Biomech Eng 121(6): 657-62.

Merican AM, Amis AA (2009) Iliotibial band tension affects patellofemoral and tibiofemoral kinematics. J Biomech 42(10): 1539-46. doi: 10.1016/j.jbiomech.2009.03.041

Mesfar W, Shirazi-Adl A (2006) Biomechanics of changes in ACL and PCL material properties or prestrains in flexion under muscle force-implications in ligament reconstruction. Comput Methods Biomech Biomed Engin 9(4): 201-9. doi: 10.1080/10255840600795959

Miller Z, Fuchs MB, Arcan M (2002) Trabecular bone adaptation with an orthotropic material model. J Biomech 35(2): 247-56.

Morgan EF, Bayraktar HH, Keaveny TM (2003) Trabecular bone modulus-density relationships depend on anatomic site. J Biomech 36(7): 897-904. doi: 10.1016/S0021-9290(03)00071-X

Phillips ATM (2009) The femur as a musculo-skeletal construct: a free boundary condition modelling approach. Med Eng Phys 31(6): 673-80. doi: 10.1016/j.medengphy.2008.12.008

Phillips ATM, Villette CC, Modenese L (2015) Femoral bone mesoscale structural architecture prediction using musculoskeletal and finite element modelling. International Biomechanics 2(1): 43-61. doi: 10.1080/23335432.2015.1017609

Polgar K, Gill HS, Viceconti M, Murray DW, O'Connor JJ (2003) Strain Distribution Within the Human Femur Due to Physiological and Simplified Loading: Finite Element Analysis Using the Muscle Standardized Femur Model. Proceedings of the Institution of Mechanical Engineers Part H, Journal of engineering in medicine 217(3): 173-189.

Quental C, Folgado J, Fernandes PR, Monteiro J (2014) Subject-specific bone remodelling of the scapula. Comput Methods Biomech Biomed Engin 17(10): 1129-43. doi: 10.1080/10255842.2012.738198

Speirs AD, Heller MO, Duda GN, Taylor WR (2007) Physiologically based boundary conditions in finite element modelling. J Biomech 40(10): 2318-23. doi: 10.1016/j.jbiomech.2006.10.038

Staubli HU, Schatzmann L, Brunner P, Rincon L, Nolte LP (1996) Quadriceps tendon and patellar ligament: cryosectional anatomy and structural properties in young adults. Knee surgery, sports traumatology, arthroscopy : official journal of the ESSKA 4(2): 100-10. doi: 10.1007/bf01477262

Taylor ME, Tanner KE, Freeman MAR, Yettram AL (1996) Stress and strain distribution within the intact femur: Compression or bending. Medical Engineering & Physics 18(2): 122-131. doi: Doi 10.1016/1350-4533(95)00031-3

Zajac FE (1989) Muscle and tendon: properties, models, scaling, and application to biomechanics and motor control. Crit Rev Biomed Eng 17(4): 359-411.
